# The Enhanced Inhibitory Effect of Estrogen on PD-L1 Expression Following Nrf2 Deficiency in the AOM/DSS Model of Colitis-Associated Cancer

**DOI:** 10.3389/fonc.2021.679324

**Published:** 2021-07-08

**Authors:** Changhee Kang, Chin-Hee Song, Nayoung Kim, Ryoung Hee Nam, Soo In Choi, Jeong Eun Yu, Heewon Nho, Jin A. Choi, Jin Won Kim, Hee Young Na, Ha-Na Lee, Young-Joon Surh

**Affiliations:** ^1^ Departments of Internal Medicine, Seoul National University Bundang Hospital, Seongnam, South Korea; ^2^ Department of Internal Medicine and Liver Research Institute, Seoul National University College of Medicine, Seoul, South Korea; ^3^ Departments of Pathology, Seoul National University Bundang Hospital, Seongnam, South Korea; ^4^ Laboratory of Immunology, Division of Biotechnology Review and Research-III, Office of Biotechnology Products, Center for Drug Evaluation and Research, Food and Drug Administration, Silver Spring, MD, United States; ^5^ Department of Molecular Medicine and Biopharmaceutical Sciences, Graduate School of Convergence Science and Technology, Seoul National University, Seoul, South Korea; ^6^ Cancer Research Institute, Seoul National University, Seoul, South Korea

**Keywords:** 17β-estradiol, Nrf2, programmed death-ligand 1, inflammation, immune microenvironment

## Abstract

Nuclear factor erythroid 2-related factor 2 (Nrf2) plays a dual role in carcinogenesis. We previously reported that Nrf2 deficiency enhances the anti-tumorigenic effect of 17β-estradiol (E2) in an azoxymethane (AOM)/dextran sodium sulfate (DSS) model of colitis-associated cancer (CAC). Herein, we aimed to determine a possible explanation for our recent work and investigated the immune microenvironment represented by programmed death-ligand 1 (PD-L1) expression. One week after the AOM injection, mice were administered with DSS in drinking water for seven days; daily E2 injections were intraperitoneally administered during this period. The mice were sacrificed 16 weeks after AOM injection and analyzed for PD-L1 expression in the distal colon tissues using Western blotting and immunohistochemistry (IHC). Based on Western blotting results, PD-L1 expression was reduced in Nrf2 knockout (KO) female and E2-treated male mice when compared with their wild-type counterparts, following AOM/DSS treatment; this supports the association of PD-L1 expression with tumor progression. Additionally, this finding was in good agreement with the IHC results for PD-L1. Furthermore, we observed that PD-L1 is predominantly expressed in stromal cells rather than on epithelial cells in the colon. Western blotting revealed that PD-L1 expression in the colon positively correlates with expressions of inducible nitric oxide synthase (iNOS) (male, *P* = 0.002; female, *P <*0.001) and cyclooxygenase-2 (COX-2) (male, *P <*0.001; female, *P <*0.001). Collectively, our findings indicate that estrogen ameliorates the immune microenvironment represented by PD-L1 expression and enhances its effect in the absence of Nrf2.

## Introduction

Inflammation is known to predispose patients to cancer initiation and development (1). For instance, inflammatory diseases such as chronic inflammatory bowel disease, chronic hepatitis, and *Helicobacter pylori*-induced gastritis have been associated with an increased risk of cancer (2). Tumor initiation occurs as a result of genetic mutations induced by extrinsic mutagens or inherent DNA copying errors. Inflammatory responses lead to the accumulation of mutations even in the absence of extrinsic mutagens (3). For example, activated macrophages and neutrophils produce reactive oxygen species (ROS) and reactive nitrogen species, which induce mutations in normal tissues (1). Therefore, an inadequately resolved chronic inflammation can increase the risk of cancer.

A programmed death 1 (PD-1) is an immune checkpoint expressed on activated T cells, and its engagement with programmed death-ligand 1 (PD-L1) attenuates T cell activation (4). PD-L1 expression in tumors or infiltrating immune cells plays a role in suppressing T cell activation, thereby dampening T cell cytotoxicity and further anti-tumor immune responses (5, 6). Indeed, immune checkpoint inhibitors blocking the PD-1/PD-L1 interaction have shown remarkable results in cancer therapy (7). Most recently, the therapeutic effect of trastuzumab deluxtecan (DS-8201), which is an antibody–drug conjugate, was reported in patients with CRC (8). It showed strong and durable anti-tumor activity in patients with HER2-positive metastatic CRC following two or more previous therapies (8). Furthermore, PD-L1 is expressed in various normal tissues and maintains immune homeostasis (9). For instance, PD-L1-deficient mice show an exacerbated inflammatory response in the colon, suggesting a role for PD-L1 in immune tolerance (10). Studies have revealed that induction of PD-L1 expression can occur in response to inflammatory cytokines such as interferon-gamma (IFN-γ), interleukin-17 (IL-17), and tumor necrosis factor-alpha (TNF-α) (11). Among downstream signaling pathways of inflammation, nuclear factor-κB (NF-κB) has emerged as a key regulator of PD-L1 expression (12). Therefore, chronic inflammation is closely linked to the mechanism of tumor immune evasion (12).

It has been reported that estrogen suppresses intestinal inflammation in clinical and experimental studies [reviewed in (13)], and further colitis-associated cancer (CAC) development in an azoxymethane (AOM)/dextran sodium sulfate (DSS) mouse model (14, 15). Conversely, several studies have revealed that estrogen facilitates tumorigenesis by promoting immunosuppression (e.g. increasing PD-L1 expression on tumor cells) in the tumor microenvironment (TME) (16–18). However, in these studies, the effects of estrogen were evaluated on existing tumor cells. Estrogen receptors (ERs) are classified as ERα and ERβ, both of which have distinct roles: that ERα is oncogenic and promotes cellular proliferation, whereas ERβ is predominantly protective, being anti-proliferative, anti-inflammatory, and anti-tumorigenic (19). In the previous study, 17β-estradiol (E2) treatment induced the expression of ERβ but not ERα in CCD841CoN cells, a female human colonic epithelial cell line. E2 treatment significantly elevated the expression of anti-oxidant enzymes such as heme oxygenase-1 (HO-1) and NAD(P)H-quinone oxidoreductase-1 (NQO1) (20). Furthermore, E2 supplementation decreased protein and mRNA levels of NF-kB-related pro-inflammatory enzymes such as nitric oxide synthase (iNOS) and cyclooxygenase-2 (COX-2) induced by AOM/DSS treatment in the colitis stage (14). These results suggest that estrogen exerts its anti-inflammatory and anti-oxidant effects *via* ERβ (20).

Nuclear factor erythroid 2-related factor 2 (Nrf2) transcription factor maintains redox homeostasis during oxidative stress and suppresses inflammation (21). Consistent with its role, Nrf2 deficiency exacerbates inflammation in various murine models (21). Accordingly, it has been established that Nrf2 plays a role in suppressing the inflammatory response, thus protecting cells from inflammation-induced carcinogenesis (21). Furthermore, we have previously revealed that Nrf2 can mediate the anti-inflammatory effect of E2 in mouse embryonic fibroblast cells (22). This led us to postulate that the effect of E2 would be impaired in Nrf2-deficient mice. However, our recent study showed that the anti-tumorigenic effect of E2 was more prominent in Nrf2 knockout (KO) male mice than in wild-type (WT) male mice (23). These results indicate that Nrf2 activation may not be beneficial under certain conditions. Moreover, Nrf2 activation protects not only normal cells but also cancer cells from oxidative stress which promotes cancer progression (24). More importantly, a recent study has reported that Nrf2 transcriptionally regulates PD-L1 expression (25). In tumor-bearing mice, Nrf2 deficiency decreases the tumor burden and inhibits the function and survival of myeloid-derived suppressor cells (MDSCs) (26), which express high levels of PD-L1 and contribute to the immunosuppressive TME (27).

Based on this background, we hypothesized that estrogen might exert anti-tumorigenic effects and cooperate with Nrf2 deficiency in the immune microenvironment Therefore, this study aimed to determine the sex difference in PD-L1 expression and its correlation with Nrf2 expression in the colon tumor microenvironment of the AOM/DSS-induced CRC mouse model. Also, as part of the sex difference, we tried to confirm the effect of E2 on PD-L1 expression in male mice.

## Material and Methods

### Reagents

AOM (# A5486) and E2 (# E8876) were purchased from Sigma-Aldrich (St. Louis, MO, USA). DSS (molecular weight 36,000–50,000, # 160110) was procured from MP Biomedicals (Aurora, OH, USA). The antibodies used in this study are listed in **Table 1**.

**Table 1:** The antibody list for Western blotting and immunohistochemistry.

Antibody	Applications	Dilution	Company	Catalog number
PD-L1	WB	1:2,000	Abcam	ab213480
iNOS	WB	1:4,000	Cayman	160862
COX-2	WB	1:3,000	Cayman	160106
Nrf2	WB	1:2,000	Cell Signaling	12721
GAPDH	WB	1:2,000	Santa Cruz	sc-32233
PD-L1	IHC	1:50	eBioscience	14-5982-82

### Animals

Heterozygous Nrf2^+/−^ mice (C57BL/6 background) were kindly provided by Prof. Young-Joon Surh. As shown in **Supplementary Figures S1A, B**, littermates of WT and Nrf2 knockout (Nrf2^−/−^, KO) were generated by cross-breeding of heterozygous Nrf2^+/−^ mice (23, 28). Mice were genotyped by performing a tail DNA extraction, followed by a polymerase chain reaction. Bands were detected by agarose gel electrophoresis at 214 bp for Nrf2^−/−^ and 263 bp for WT mice, as detailed in our previous study (23, 28). All animals were maintained under a 12-hour light/dark cycle and allowed water and food *ad libitum*. Animal care and experiments were performed in accordance with the Animal Research: Reporting of *In Vivo* Experiments (ARRIVE) guidelines. All animal experiment protocols were approved by the Institutional Animal Care and Use Committee (IACUC) of the Seoul National University Bundang Hospital (BA1705-223/043-01).

### AOM/DSS Model of Colitis-Associated Cancer

**Figure 1:**
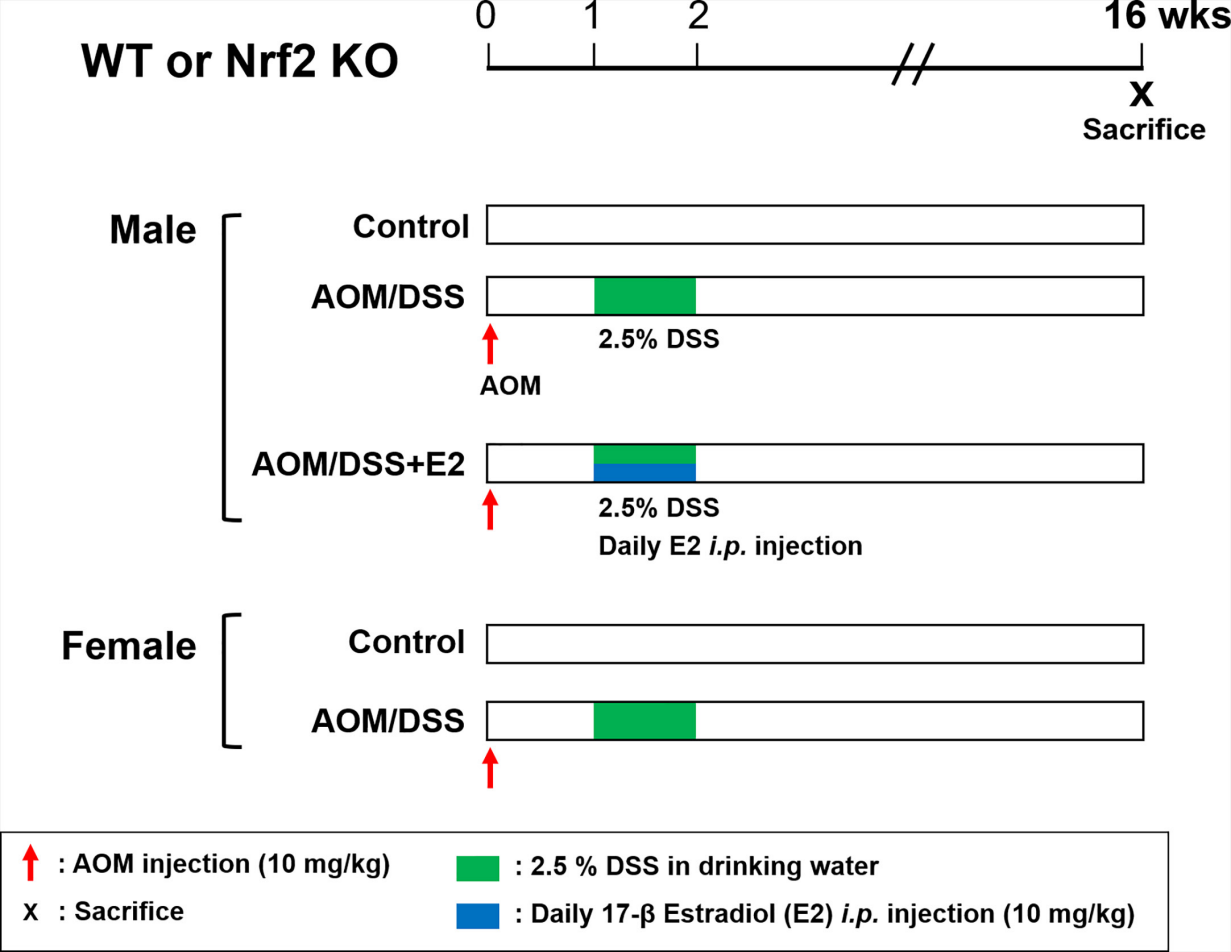
Overview of the experimental design. WT and Nrf2 KO male and female mice were used in the AOM/DSS-induced colitis-associated CRC protocol. AOM (10 mg/kg) was injected into the mice on Day 0. One week after, DSS (2.5%) was provided in the drinking water for one week. E2 (10 mg/kg) was administrated by daily intraperitoneal injection for one week during DSS treatment. The mice were sacrificed at week 16 after AOM injection. WT, wild-type; Nrf2, nuclear factor erythroid 2–related factor 2; KO, knockout; AOM, azoxymethane; DSS, dextran sodium sulfate; E2, 17β-estradiol.

The AOM/DSS protocol is well established and is commonly used as a mouse model of CAC. Both WT and Nrf2 KO mice were randomly divided into the following five groups: male control group, male AOM/DSS group, male AOM/DSS + E2 group, female control group, and female AOM/DSS group; each group was composed of 9–12 mice. In the AOM/DSS-treated group, mice were intraperitoneally injected with 10 mg/kg of AOM dissolved in phosphate-buffered saline (PBS). Seven days after injection, 2.5% (w/v) DSS was added to the drinking water for 7 days, followed by switching to regular water. E2 was dissolved in olive oil and administered intraperitoneally daily at a dose of 10 mg/kg during 2.5% DSS treatment (**Figure 1**). After 16 weeks, all mice were sacrificed, and distal colon tissues were processed for further analysis. Colon samples were derived from the same tissue samples used in our previous experiments (23, 28).

### Western Blot Analysis

Colon samples were homogenized using a handheld homogenizer in 300 μl of RIPA buffer supplemented with complete protease inhibitors (Roche, South San Francisco, CA). Then, tissue lysates were denatured and separated using sodium dodecyl sulfate-polyacrylamide gel electrophoresis (SDS-PAGE). Following electrophoresis, separated proteins were transferred to polyvinylidene difluoride (PVDF) membranes. The membranes were blocked with 5% bovine serum albumin (BSA) in TBST (20 mM Tris, 150 mM NaCl, containing 0.1% Tween-20, pH 7.4) for 1 h. Next, the membranes were incubated with primary antibodies (**Table 1**) in TBST with 5% BSA at 4°C overnight. After removing the excess primary antibody by washing, the membranes were incubated with horseradish peroxidase (HRP)-conjugated secondary antibody at room temperature for 1 h. After washing, the membranes were exposed to enhanced chemiluminescence (ECL) reagent and visualized using a ChemiDoc MP (Bio-Rad). Detection and quantification of band intensities were performed using Image Lab 5.0 software (Bio-Rad).

### Quantitative Real-Time Polymerase Chain Reaction

Colon tissues were homogenized using a handheld homogenizer in 500 μl of TRIzol reagent (Invitrogen, Carlsbad, CA, USA), and total RNA was extracted. According to the manufacturer’s instructions, 2 μg of total RNA was reverse transcribed using a Reverse Transcription kit (Applied Biosystems, Foster City, CA, USA). The cDNA was used to perform qRT-PCR using specific primers (listed in **Table 2**) in a QuantStudio™ 7 Flex Real-Time PCR instrument. The expression levels were normalized to that of *Gapdh*.

**Table 2:** List of oligonucleotide sequences used for qRT-PCR.

Gene	Sequence (5’➔3’)
*Pd-l1*	F: TGC GGA CTA CAA GCG AAT CAC G R: CTC AGC TTC TGG ATA ACC CTC G
*iNos*	F: TGG TGG TGA CAA GCA CAT TT R: AAG GCC AAA CAC AGC ATA CC
*Ho-1*	F: CCT CAC TGG CAG GAA ATC ATC R: CCT CGT GGA GAC GCT TTA CAT A
*Nqo1*	F: GCG AGA AGA GCC CTG ATT GTA CTG R: TCT CAA ACC AGC CTT TCA GAA TGG
*Gaphd*	F: TTC ACC ACC ATG GAG AAG GC R: GGC ATG GAC TGT GGT CAT GA

### Immunohistochemistry

Colon tissues were fixed with phosphate-buffered formalin and embedded in paraffin. Tissue sections were blocked with 3% hydrogen peroxide and normal goat serum. For PD-L1, immunohistochemical staining was performed using the MIH5 rat monoclonal antibody (eBioscience, San Diego, USA) on a Benchmark XT autostainer (Ventana Medical Systems, Tucson, AZ, USA), with standard antigen retrieval. The UltraView Universal DAB Detection Kit (Ventana Medical Systems) was used according to the manufacturer’s instructions. PD-L1 expression was evaluated in both the tumor and non-tumor areas. Quantitative assessment of PD-L1 immunoreactivity was performed using the Image-Pro Plus analysis system, with measurements expressed as the proportion of immunoreactive area (% of total area). The reported values are averages of different images from different mice within each group.

### Statistical Analysis

Statistical analysis was performed using SPSS version 18.0 (SPSS, Chicago, IL, USA). Data are presented in bar graphs as the mean ± standard error of the mean (SEM). The statistical significance of differences between the two groups was evaluated using the Mann–Whitney test, and the graphs were generated using GraphPad Prism 5.0 (GraphPad Software, San Diego, USA). The statistical significance of differences between more than two groups was determined using a nonparametric factor Kruskal–Wallis sum-rank test. Statistical significance was set at *P <*0.05. The correlation of PD-L1 expression with COX-2 and iNOS was determined using Spearman’s correlation coefficient.

## Results

### Strong Inhibitory Effect of Estrogen on the Expression of PD-L1 And Inflammatory Mediators in the Absence of Nrf2

**Figure 2:**
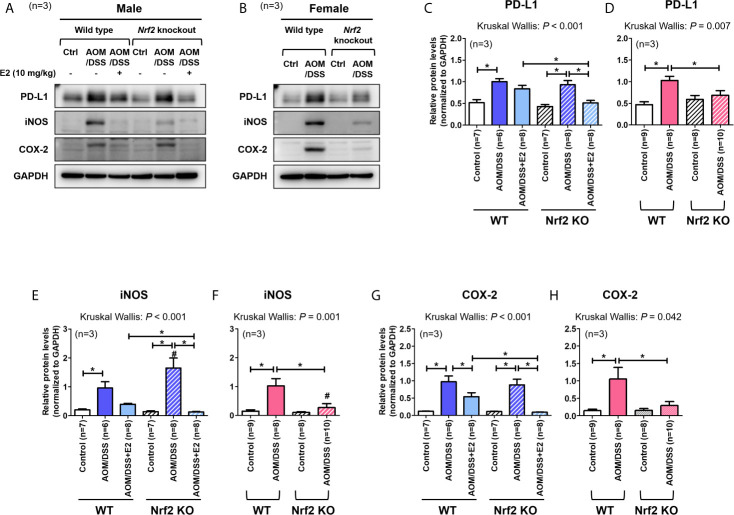
Stronger inhibitory effect of E2 on AOM/DSS-induced PD-L1 expression in Nrf2 KO male mice than in WT males. **(A, B)** Representative Western blot images of three independent experiments performed with colon tissue from the male **(A)** and female mice **(B)**. **(C–H)** Relative protein expression of PD-L1 **(C, D)**, iNOS **(E, F)**, and COX-2 **(G, H)** in males **(C, E, G)** and females **(D, F, H)**. GAPDH was used as a loading control. The *P*-values obtained from the Kruskal–Wallis test is shown in the figure; *P < 0.05 for comparison between two groups (Mann–Whitney test); ^#^ *P <* 0.05 for comparison between males and females (Mann–Whitney test). WT, wild-type; KO, knockout; Ctrl, control; AOM, azoxymethane; DSS, dextran sodium sulfate; PD-L1, programmed death-ligand 1; iNOS, inducible nitric oxide synthase; COX-2, cyclo-oxygenase 2; Nrf2, nuclear factor erythroid 2-related factor 2; GAPDH, Glyceraldehyde 3-phosphate dehydrogenase.

We have previously reported that E2 inhibits colitis-associated tumorigenesis (14) and that Nrf2 deficiency enhances the anti-tumorigenic effect of E2 (23) in an AOM/DSS model of CAC. Herein, we examined whether the anti-tumorigenic effect of E2 is associated with PD-L1 expression in distal colon tissues in the same model. The colon tissues used in this study were derived from tissues used in our previous study (23), and **Figure 1** depicts the experimental procedure. After preferentially confirming the expression of Nrf2 in colon tissues of WT and Nrf2 KO groups (**Supplementary Figure S1C**), the protein expression of PD-L1 and pro-inflammatory mediators, including iNOS and COX-2, in colon tissues were determined by Western blot analysis. As shown in **Figures 2A, C**, AOM/DSS treatment significantly increased PD-L1 protein expression in WT (*P* = 0.003) and Nrf2 KO (*P* = 0.004) male mice when compared with the control group. The administration of E2 significantly reduced the expression of AOM/DSS-induced PD-L1 protein in Nrf2 KO male mice (*P* = 0.009); a marginal decrease was observed in WT male mice when compared with that in the AOM/DSS group (**Figures 2A, C**). We further evaluated the E2 effects on the protein levels of iNOS and COX-2 in the presence or absence of Nrf2. Consistent with previous results (23), AOM/DSS-induced iNOS protein levels were significantly decreased by E2 administration only in Nrf2 KO males (*P <*0.001), but not in WT males (**Figures 2A, E**). Also, COX-2 protein levels induced by AOM/DSS treatment were significantly decreased by E2 administration both in WT (*P* = 0.004) and Nrf2 KO (*P <*0.001) males (**Figures 2A, G**). These results revealed that E2 reduces PD-L1 expression along with iNOS and COX-2 expression more effectively in the absence of Nrf2, suggesting its protective role of E2 in the immune microenvironment and tumorigenesis.

A previous report has revealed that Nrf2-deficient male mice are more susceptible to AOM/DSS-induced intestinal inflammation and tumorigenesis than WT male mice (29). In contrast, our studies using female mice showed that Nrf2 deficiency significantly attenuated tumorigenesis (based on the number of tumors larger than 2 mm) induced by AOM/DSS treatment (28). Therefore, we aimed to determine whether PD-L1 protein expression in colon tissues differs between WT and Nrf2 KO female mice following exposure to AOM/DSS. As shown in **Figures 2B, D**, AOM/DSS treatment significantly increased PD-L1 protein expression in WT females (*P <*0.001), but not in Nrf2 KO females. Similar to PD-L1 expression, iNOS and COX-2 protein levels were strongly elevated by AOM/DSS treatment only in WT females (*P* = 0.002 for iNOS and *P* = 0.018 for COX-2), not in Nrf2 KO females (**Figures 2B, F, H**). Unlike their male counterparts, female mice lacking Nrf2 presented significantly lower expression levels of PD-L1 (*P* = 0.033) and pro-inflammatory mediators (*P* = 0.003 for iNOS and *P* = 0.045 for COX-2) than WT mice after AOM/DSS treatment (**Figures 2B, D**). Furthermore, the protein levels of iNOS induced by AOM/DSS treatment were strongly lower in female mice than in male, only in Nrf2 KO groups (*P* = 0.004 for male *vs* female in Nrf2 KO AOM/DSS group; **Figures 2E, F**), not in WT.

**Figure 3:**
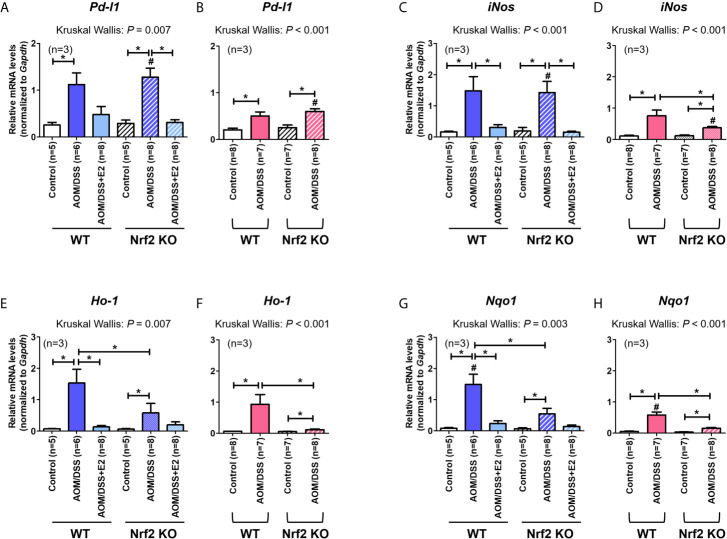
Effect of E2 on mRNA expression of *Pd-l1*, *iNos*, and Nrf2-mediated anti-oxidant enzyme genes. **(A–H)** mRNA expression of *Pd-l1* **(A, B)**, *iNos* **(C, D)**, *Ho-1* **(E, F)**, and *Nqo1* **(G, H)** in WT and Nrf2 KO male **(A, C, E, G)** and female **(B, D, F, H)** mice. *Gapdh* was used as an internal control. The *P*-values obtained from the Kruskal–Wallis test is shown in the figure; *P < 0.05 for comparison between two groups (Mann–Whitney test); ^#^ *P <* 0.05 for comparison between males and females (Mann–Whitney test). WT, wild-type; KO, knockout; AOM, azoxymethane; DSS, dextran sodium sulfate; *Pd-l1*, programmed death-ligand 1; *iNos*, inducible nitric oxide synthase; *Ho-1*, heme oxygenase 1; *Nqo1*, NAD(P)H dehydrogenase (quinone) 1; *Gapdh*, Glyceraldehyde 3-phosphate dehydrogenase.

Next, we analyzed the mRNA expression of *Pd-l1* and *iNos*. Similar to protein levels, the mRNA expression of *Pd-l1* and *iNos* increased by AOM/DSS treatment was more strongly suppressed by E2 treatment in Nrf2 KO males than in WT males (**Figures 3A, C**). In female mice, AOM/DSS-induced *iNos* expression was significantly lower in Nrf2 KO mice compared with WT mice (*P* = 0.049). In terms of sex, the mRNA expression of *Pd-l1* and *iNos* increased by AOM/DSS treatment was significantly lower in female mice than in males, only in Nrf2 KO groups (*P* = 0.012 for *Pd-l1* and *P* = 0.009 for *iNos*; **Figures 3A–D**). We further evaluated the mRNA expression of Nrf2-mediated anti-oxidant enzyme genes, including *Ho-1* and *Nqo1*. In WT males, the mRNA expression of *Ho-1* and *Nqo1*, which was strongly increased by AOM/DSS treatment, was strongly inhibited by E2 treatment (*P* = 0.005 for *Ho-1* and *P* = 0.010 for *Nqo1*; **Figures 3E, G**). Contrary to WT male mice, the inhibitory effect of E2 on *Ho-1* and *Nqo1* mRNA expression increased by AOM/DSS treatment was weak in the Nrf2 KO males (**Figures 3E, G**). Furthermore, *Ho-1* and *Nqo1* mRNA expressions increased by AOM/DSS treatment were significantly lower in Nrf2 KO compared with WT in both males and females (*P* = 0.039 for *Ho-1* and *P* = 0.020 for *Nqo1* in males; *P* = 0.003 for *Ho-1* and *P* = 0.001 for *Nqo1* in females; **Figures 3E–H**). Accordingly, the sexual dimorphism of Nrf2 deficiency can be observed in PD-L1 expression in response to AOM/DSS, suggesting an association between PD-L1 expression and tumorigenesis.

### Correlation Between PD-L1 and Pro-Inflammatory Mediators in the Colon Tumor Microenvironment

**Figure 4:**
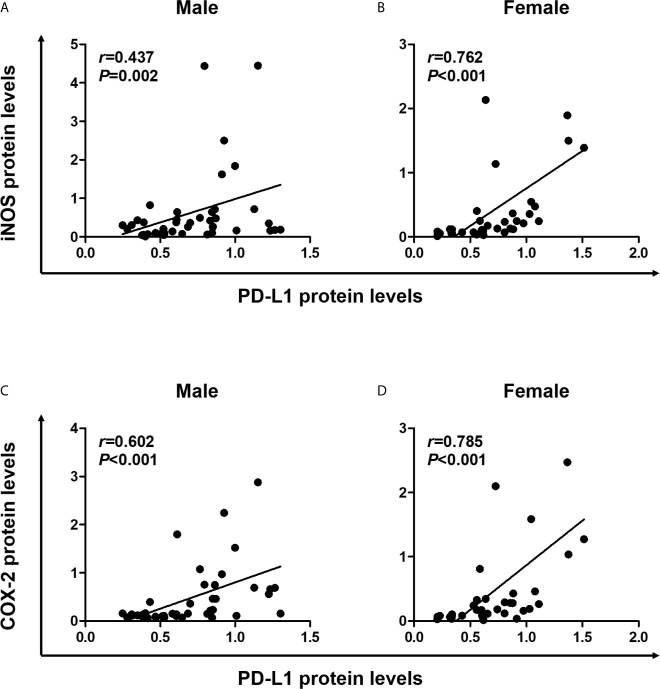
Positive correlation of PD-L1 expression with proinflammatory mediators. **(A, B)** Correlation of PD-L1 protein levels with iNOS protein levels in male **(A)** and female **(B)** mice. **(C, D)** Correlation of PD-L1 protein levels with COX-2 protein levels in male **(C)** and female **(D)** mice. Spearman correlation analysis was used to determine the correlation of results from the Western blotting (**Figure 2**). PD-L1, programmed death-ligand 1; iNOS, inducible nitric oxide synthase; COX-2, cyclooxygenase 2.

Pro-inflammatory cytokines produced in the TME induce PD-L1 expression in tumor cells (11), and the inflammatory milieu recruits PD-L1-positive immune cells to the tumor sites (1). Next, we conducted statistical analysis to determine the correlation between expression levels of PD-L1 and inflammatory markers, including iNOS and COX-2 in colon tissues. In male mice, Spearman correlation analysis revealed that PD-L1 expression significantly correlated with the expressions of iNOS (*P* = 0.002) and COX-2 (*P <*0.001) (**Figures 4A, C**). Furthermore, similar to results observed in male mice, PD-L1 expression positively correlated with expressions of iNOS (*P <*0.001) and COX-2 (*P <*0.001) in both WT and Nrf2 KO female mice (**Figures 4B, D**). These results suggest that the inflammatory milieu is closely associated with the suppressive immune microenvironment. Moreover, the protective role of E2 in tumorigenesis might be related to the suppression of iNOS and COX-2 expression.

### Frequencies of PD-L1-Positive Stromal Cells Are Lower in E2-Treated Nrf2 KO Male Mice and Nrf2 KO Female Mice When Compared With WT Mice After AOM/DSS Treatment

**Figure 5:**
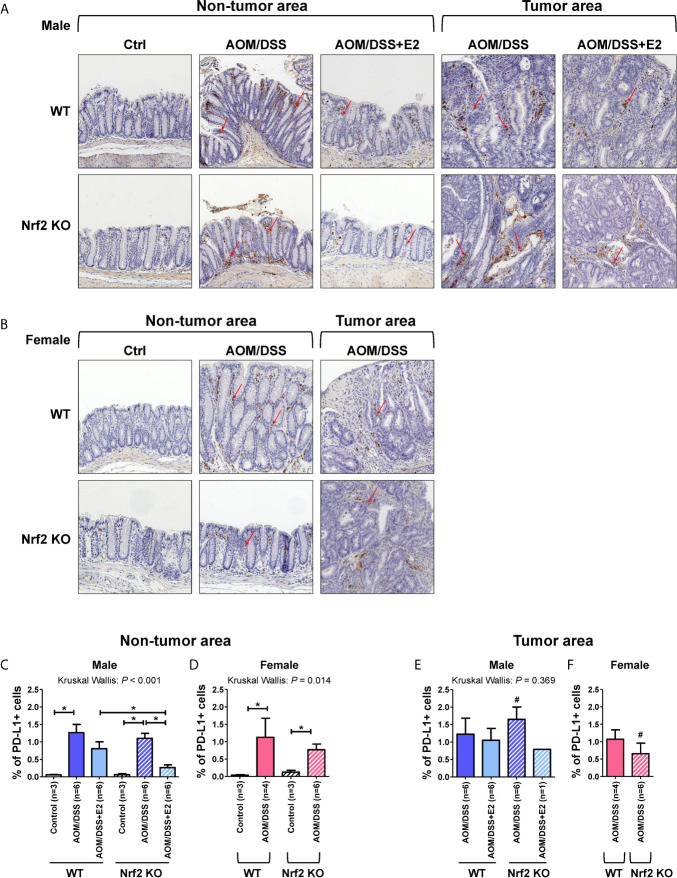
PD-L1-positive stromal cells are reduced in E2-treated Nrf2 KO male mice and Nrf2 KO female mice when compared with WT mice after AOM/DSS treatment. **(A, B)** Representative IHC images of PD-L1 expression in non-tumor and tumor areas in colon tissues of male **(A)** and female **(B)** mice. Magnification, ×200. Arrows indicate PD-L1-positive stromal cells. **(C–F)** PD-L1 expression according to the non-tumor area **(C, D)** and tumor area **(E, F)** in male **(C, E)** and female **(D, F)** mice were quantified as a percentage of the immunoreactive area (% of total area). Bar graph values are expressed as the mean ± standard error of the mean (SEM). The P-values obtained from the Kruskal–Wallis test is shown in the figure; *P < 0.05 for comparison between two groups (Mann–Whitney test); ^#^ *P* < 0.05 for comparison between males and females (Mann–Whitney test). WT, wild-type; KO, knockout; Ctrl, control; AOM, azoxymethane; DSS, dextran sodium sulfate; Nrf2, nuclear factor erythroid 2-related factor 2; PD-L1, programmed death-ligand 1.

PD-L1 can be expressed by various cells, including tumor, immune, and stromal cells. Therefore, IHC was performed on colon tissues to identify the type of PD-L1-expressing cells and to quantify those cells in the tumor and non-tumor areas, respectively, by a colon pathologist (HYN). PD-L1 expression was analyzed in male and female mice, and representative images were obtained from the indicated mice. As shown in **Figures 5A, B**, PD-L1 expression was almost exclusively observed in stromal cells and mononuclear immune cells (lymphocytes and macrophages) rather than epithelial cells, in both tumor and non-tumor areas. In male mice, PD-L1-positive cells were barely detected in the control group of WT and Nrf2 KO mice, but AOM/DSS treatment significantly increased the frequencies of PD-L1-positive cells in both tumor (*P* = 0.020 for WT and *P* = 0.020 for Nrf2 KO; **Figures 5A, E**) and non-tumor areas (*P* = 0.024 for WT and *P* = 0.024 for Nrf2 KO; **Figures 5A, C**) in colon tissues. After combined treatment with E2 and AOM/DSS, the frequency of PD-L1-positive cells decreased profoundly in Nrf2 KO male mice (*P* = 0.025) when compared with WT male mice in the non-tumor area (**Figures 5A, C**). All WT male mice co-treated with AOM/DSS and E2 developed tumors, but interestingly, only one out of six mice co-treated with AOM/DSS and E2 developed a tumor in the Nrf2 KO group (**Figures 5C, E**). The frequency of PD-L1-positive cells increased by AOM/DSS treatment in the tumor area tended to decrease by E2 treatment in the Nrf2 KO male mice than in the WT males, but did not show significance due to the low ‘N’ number of AOM/DSS + E2 group in Nrf2 KO (**Figure 5E**).

Similar to the male control group, PD-L1-positive cells were barely detectable in the controls of WT and Nrf2 KO female mice (**Figures 5B, D**). Also, AOM/DSS treatment significantly increased the frequency of PD-L1-positive cells both in tumor (*P* = 0.034 for WT and *P* = 0.020 for Nrf2 KO; **Figures 5B, F**) and non-tumor areas (*P* = 0.034 for WT and *P* = 0.020 for Nrf2 KO; **Figures 5B, D**) in WT and Nrf2 KO female mice, but to a lesser extent in Nrf2 KO female mice, when compared with WT mice; the difference was not statistically significant. In terms of sex, compared with their male counterparts, the frequencies of PD-L1-positive cells enhanced by AOM/DSS treatment in non-tumor areas were slightly lower in female mice of Nrf2 KO and WT group. Furthermore, the frequencies of PD-L1-positive cells within tumor areas were strongly lower in Nrf2 KO female mice than in Nrf2 KO males (*P* = 0.025 for male *vs* female; **Figures 5E, F**), but not in WT mice, suggesting sex differences in PD-L1 expression in response to AOM/DSS treatment. These findings corresponded with the reduced tumorigenesis in female mice when compared with that in male mice after AOM/DSS treatment (14). Moreover, PD-L1 expression evaluated by IHC showed good concordance with the Western blot results presented in **Figure 2**.

## Discussion

Previously, we reported that E2 more effectively suppressed AOM/DSS-induced tumorigenesis in Nrf2 KO male mice than in WT males (23). As a possible explanation for this finding, the present study revealed that tumor progression was associated with an increased frequency of PD-L1-positive cells in colon tissues. Furthermore, PD-L1 expression in colon tissues positively correlated with iNOS and COX-2 expressions, implying that the inflammatory milieu is involved in PD-L1 expression. These results indicate that the anti-tumorigenic effect of E2 might be associated with the immune microenvironment and cooperate with Nrf2 deficiency.

The prognostic value of PD-L1 expression in colorectal cancer (CRC) remains elusive. Recent studies using meta-analysis have shown that PD-L1 expression is a negative prognostic factor for overall survival and disease-free survival (30, 31). Although PD-L1 can be expressed on stromal, epithelial, and immune cells in CRC (32), the relative prognostic implications of these cells need to be elucidated. In the current study, we, for the first time, revealed that PD-L1 expression in colon tissues from the AOM/DSS-induced CAC mouse model was associated with colon tumor progression. Moreover, stromal and immune cells were identified as PD-L1-positive cells in colon tissue. Consistent with our findings, a recent study using the CT26 syngeneic colon tumor mouse model demonstrated that PD-L1 expression on stromal cells promoted colon cancer progression (33).

It is widely accepted that inflammation is a risk factor for carcinogenesis, as demonstrated in numerous studies (1). As a transcription factor, Nrf2 regulates the expression of anti-oxidant genes (21). Therefore, Nrf2 exerts a protective effect against inflammation-induced carcinogenesis *via* its ability to reduce ROS accumulation (24). In contrast, recent studies have shown that Nrf2 promotes cancer progression and metastasis (34). These controversial roles of Nrf2 in cancer are described in the literature as the “dual roles” of Nrf2 in cancer (24). In the current study, we showed that E2 more effectively suppressed inflammatory markers such as iNOS and COX-2, in the absence of Nrf2, in the cancer stage. It was associated with reduced tumorigenesis by E2. Therefore, our study highlights the critical role of E2, even in the absence of Nrf2, in inflammation-induced carcinogenesis.

A previous report has revealed that Nrf2-deficient male mice were more susceptible to AOM/DSS-induced tumorigenesis than their WT counterparts (29). However, in female mice, Nrf2 deficiency significantly attenuated tumorigenesis (based on the number of tumors >2 mm in diameter) caused by AOM/DSS treatment (28). Based on our findings in Nrf2 KO male and female mice, it could be surmised that endogenous estrogen in female mice recapitulated the effect of exogenous E2 in male mice following exposure to AOM/DSS. Compared with their WT counterparts, the reduced tumorigenesis in Nrf2 KO female and E2-treated Nrf2 KO male mice was accompanied by reduced expression of PD-L1 in colon tissues (**Figures 2** and **3**). Moreover, Nrf2 KO female and E2-treated Nrf2 KO male mice showed decreased expression of inflammatory markers such as iNOS and COX-2, when compared with WT mice after AOM/DSS treatment. As NF-κB reportedly regulates the expression of iNOS and COX-2, E2 may play a role in suppressing the NF-κB signaling pathway in the AOM/DSS model of CAC. NF-κB is a key transcription factor of inflammation and is closely associated with colitis-associated tumorigenesis (35, 36). However, further studies are warranted to identify molecules involved in the action of E2 in the NF-κB signaling pathway.

Several inflammatory cytokines such as IFN-γ, IL-17, and TNF-α reportedly induce PD-L1 expression on tumor cells (11), and the inflammatory milieu recruits PD-L1-positive immune cells to the tumor sites (1). In addition, NF-κB has emerged as an important regulator of PD-L1 expression in the TME (12). To evaluate inflammatory conditions affecting PD-L1 expression, we examined the expression of inflammatory markers, such as iNOS and COX-2, in colon tissues. Our findings revealed that inflammatory signaling, as shown by COX-2 and iNOS expression, positively correlates with PD-L1 expression in colon tissues. Consistently, previous studies have demonstrated that COX-2 expression is associated with PD-L1 expression and immune evasion in melanoma (37) and breast cancer (38), thereby providing a rationale for evaluating COX-2 inhibition to prevent tumor immune evasion (38). In light of tumor immune evasion, our results, for the first time, suggest that the anti-tumorigenic effects of estrogen could be utilized through the suppression of PD-L1 expression. Conversely, most studies focused on hormone-sensitive cancer have shown that estrogen has a tumor-promoting effect (39). In addition, several recent studies have revealed that, in syngeneic tumor models, estrogen promotes tumor progression *via* the accumulation of MDSCs (16, 17), which are potent immunosuppressive cells in the TME (27). These discrepancies could be attributed to different tumor models and experimental designs. Although most studies have focused on existing tumor cells to evaluate the effects of estrogen, we investigated the effects of estrogen on colon tissues in the inflammatory phase later resulting in tumor formation by employing the AOM/DSS model of CAC and Nrf2 KO mice. Our results indicate that inadequately resolved chronic inflammation can induce immunosuppression, as represented by PD-L1 expression, and that estrogen plays a vital role in ameliorating inflammation, even in the absence of Nrf2.

In addition to being expressed on tumor cells, PD-L1 is expressed on immune cells such as MDSCs, tumor-associated macrophages, regulatory T cells, and dendritic cells (11). However, the relative contribution of these cells to immunosuppression remains poorly understood. Recent studies have revealed that PD-L1 on immune cells, rather than on tumor cells, plays a more critical role and is the relevant mechanistic target for PD-1/PD-L1 inhibitors (40, 41). Therefore, we performed IHC using colon tissues to examine which cell types expressed PD-L1. We observed that PD-L1 expression was almost exclusively found in stromal and immune cells rather than in tumor cells. Moreover, PD-L1-positive cells were significantly observed in non-tumor and tumor areas. Therefore, we quantified PD-L1-positive cells in both the tumor and non-tumor areas respectively. AOM/DSS treatment increased the frequency of PD-L1-positive cells in both tumor and non-tumor areas. After the addition of E2, the frequency of PD-L1-positive cells decreased more profoundly in Nrf2 KO mice than in WT mice. Similar to male mice, AOM/DSS treatment increased the frequency of PD-L1-positive cells in female mice. These IHC results, at least in part, showed good concordance with PD-L1 expression analyzed by Western blotting. Based on these observations, we suggest that PD-L1-positive stromal and immune cells, rather than tumor cells, might contribute to tumor progression in the AOM/DSS model of CAC.

Our study had several limitations. First, Western blot analysis did not individually demonstrate PD-L1 expression in tumor and non-tumor areas; the main reason for this was the difficulty in obtaining separate tissues from the total colon. However, the results showed good concordance with the PD-L1 expression analyzed by IHC. The second limitation is that the exact types of PD-L1-positive cells were not delineated using cell type-specific markers, although various types of PD-L1-positive cells contribute to immunosuppression depending on the context of the TME. To compensate for these limitations, we plan to further conduct T cell immunology studies in the tumor state, along with the identification of cells expressing PD-L1 in colon tissues in an AOM/DSS-induced CRC mouse model. Despite these limitations, our results undoubtedly show that the frequency of PD-L1-positive cells in colon tissues is associated with disease progression in the AOM/DSS model of CAC. To further verify the therapeutic effect of estrogen in the WT and Nrf2 KO CRC mouse model induced by AOM/DSS treatment, further studies using PD-L1 antibody should be conducted. In-depth, additional experiments are needed to further validate key findings *in vitro* using colon cancer cells and dissect the crosstalk between Nrf2 and PD-L1. In conclusion, our study highlights the critical role of estrogen in inflammation and PD-L1 expression, which cooperates with Nrf2 deficiency to suppress tumorigenesis.

## Data Availability Statement

The original contributions presented in the study are included in the article/**Supplementary Material**. Further inquiries can be directed to the corresponding author.

## Ethics Statement

The animal study was reviewed and approved by Institutional Animal Care and Use Committee (IACUC) of the Seoul National University Bundang Hospital.

## Author Contributions

CK drafted the manuscript and performed molecular experiments. C-HS revised the manuscript and performed molecular experiments. NK designed and supervised the experiments and revised the manuscript. RN, C-HS, SC, JY, and HN performed the animal experiments. JK advised on the concept of PD-L1 in Nrf2 knockout mice. HYN assisted with the analysis of immunohistochemistry of PD-L1. RN performed the immunohistochemical analysis. JC and H-NL revised the manuscript. Y-JS provided Nrf2^+/−^ transgenic mice and revised the manuscript. All authors contributed to the article and approved the submitted version.

## Funding

This work was supported by a grant from the National Research Foundation of Korea (NRF) funded by the government of the Republic of Korea (2019R1A2C2085149).

## Conflict of Interest

The authors declare that the research was conducted in the absence of any commercial or financial relationships that could be construed as a potential conflict of interest.

## References

[1] Greten FR Grivennikov SI. Inflammation and Cancer: Triggers, Mechanisms, and Consequences. Immunity (2019) 51(1):27–41. 10.1016/j.immuni.2019.06.025 31315034 PMC6831096

[2] Trinchieri G. Cancer and Inflammation: An Old Intuition With Rapidly Evolving New Concepts. Annu Rev Immunol (2012) 30:677–706. 10.1146/annurev-immunol-020711-075008 22224761

[3] Meira LB Bugni JM Green SL Lee CW Pang B Borenshtein D. DNA Damage Induced by Chronic Inflammation Contributes to Colon Carcinogenesis in Mice. J Clin Invest (2008) 118(7):2516–25. 10.1172/jci35073 PMC2423313 18521188

[4] Freeman GJ Long AJ Iwai Y Bourque K Chernova T Nishimura H. Engagement of the PD-1 Immunoinhibitory Receptor by a Novel B7 Family Member Leads to Negative Regulation of Lymphocyte Activation. J Exp Med (2000) 192(7):1027–34. 10.1084/jem.192.7.1027 PMC2193311 11015443

[5] Juneja VR McGuire KA Manguso RT LaFleur MW Collins N Haining WN. PD-L1 on Tumor Cells is Sufficient for Immune Evasion in Immunogenic Tumors and Inhibits CD8 T Cell Cytotoxicity. J Exp Med (2017) 214(4):895–904. 10.1084/jem.20160801 28302645 PMC5379970

[6] Lau J Cheung J Navarro A Lianoglou S Haley B Totpal K. Tumour and Host Cell PD-L1 is Required to Mediate Suppression of Anti-Tumour Immunity in Mice. Nat Commun (2017) 8:14572. 10.1038/ncomms14572 28220772 PMC5321797

[7] Gong J Chehrazi-Raffle A Reddi S Salgia R. Development of PD-1 and PD-L1 Inhibitors as a Form of Cancer Immunotherapy: A Comprehensive Review of Registration Trials and Future Considerations. J Immunother Cancer (2018) 6(1):8. 10.1186/s40425-018-0316-z 29357948 PMC5778665

[8] Siena S Di Bartolomeo M Raghav K Masuishi T Loupakis F Kawakami H. Trastuzumab Deruxtecan (DS-8201) in Patients With HER2-Expressing Metastatic Colorectal Cancer (DESTINY-CRC01): A Multicentre, Open-Label, Phase 2 Trial. Lancet Oncol (2021) 22(6):779–89. 10.1016/S1470-2045(21)00086-3 33961795

[9] Qin W Hu L Zhang X Jiang S Li J Zhang Z. The Diverse Function of PD-1/PD-L Pathway Beyond Cancer. Front Immunol (2019) 10:2298. 10.3389/fimmu.2019.02298 31636634 PMC6787287

[10] Scandiuzzi L Ghosh K Hofmeyer KA Abadi YM Lázár-Molnár E Lin EY. Tissue-Expressed B7-H1 Critically Controls Intestinal Inflammation. Cell Rep (2014) 6(4):625–32. 10.1016/j.celrep.2014.01.020 PMC3962725 24529703

[11] Sun C Mezzadra R Schumacher TN. Regulation and Function of the PD-L1 Checkpoint. Immunity (2018) 48(3):434–52. 10.1016/j.immuni.2018.03.014 PMC7116507 29562194

[12] Antonangeli F Natalini A Garassino MC Sica A Santoni A Di Rosa F. Regulation of PD-L1 Expression by NF-κb in Cancer. Front Immunol (2020) 11:584626. 10.3389/fimmu.2020.584626 33324403 PMC7724774

[13] Nie X Xie R Tuo B. Effects of Estrogen on the Gastrointestinal Tract. Dig Dis Sci (2018) 63(3):583–96. 10.1007/s10620-018-4939-1 29387989

[14] Son HJ Sohn SH Kim N Lee HN Lee SM Nam RH. Effect of Estradiol in an Azoxymethane/Dextran Sulfate Sodium-Treated Mouse Model of Colorectal Cancer: Implication for Sex Difference in Colorectal Cancer Development. Cancer Res Treat (2019) 51(2):632–48. 10.4143/crt.2018.060 PMC6473282 30064198

[15] Song CH Kim N Lee SM Nam RH Choi SI Kang SR. Effects of 17β-Estradiol on Colorectal Cancer Development After Azoxymethane/Dextran Sulfate Sodium Treatment of Ovariectomized Mice. Biochem Pharmacol (2019) 164:139–51. 10.1016/j.bcp.2019.04.011 30981879

[16] Milette S Hashimoto M Perrino S Qi S Chen M Ham B. Sexual Dimorphism and the Role of Estrogen in the Immune Microenvironment of Liver Metastases. Nat Commun (2019) 10(1):5745. 10.1038/s41467-019-13571-x 31848339 PMC6917725

[17] Svoronos N Perales-Puchalt A Allegrezza MJ Rutkowski MR Payne KK Tesone AJ. Tumor Cell-Independent Estrogen Signaling Drives Disease Progression Through Mobilization of Myeloid-Derived Suppressor Cells. Cancer Discovery (2017) 7(1):72–85. 10.1158/2159-8290.Cd-16-0502 27694385 PMC5222699

[18] Yang L Huang F Mei J Wang X Zhang Q Wang H. Posttranscriptional Control of PD-L1 Expression by 17β-Estradiol via PI3K/AKT Signaling Pathway in Erα-Positive Cancer Cell Lines. Int J Gynecol Cancer (2017) 27(2):196–205. 10.1097/igc.0000000000000875 27870715 PMC5258765

[19] Caiazza F Ryan EJ Doherty G Winter DC Sheahan K. Estrogen Receptors and Their Implications in Colorectal Carcinogenesis. Front Oncol (2015) 5:19. 10.3389/fonc.2015.00019 25699240 PMC4313613

[20] Son HJ Kim N Song CH Lee SM Lee HN Surh YJ. 17β-Estradiol Reduces Inflammation and Modulates Antioxidant Enzymes in Colonic Epithelial Cells. Korean J Intern Med (2020) 35(2):310–9. 10.3904/kjim.2018.098 PMC7061017 30336658

[21] Ahmed SM Luo L Namani A Wang XJ Tang X. Nrf2 Signaling Pathway: Pivotal Roles in Inflammation. Biochim Biophys Acta Mol Basis Dis (2017) 1863(2):585–97. 10.1016/j.bbadis.2016.11.005 27825853

[22] Song CH Kim N Kim DH Lee HN Surh YJ. 17-β Estradiol Exerts Anti-Inflammatory Effects Through Activation of Nrf2 in Mouse Embryonic Fibroblasts. PloS One (2019) 14(8):e0221650. 10.1371/journal.pone.0221650 31442293 PMC6707591

[23] Song CH Kim N Hee Nam R In Choi S Hee Son J Eun Yu J. 17β-Estradiol Strongly Inhibits Azoxymethane/Dextran Sulfate Sodium-Induced Colorectal Cancer Development in Nrf2 Knockout Male Mice. Biochem Pharmacol (2020) 182:114279. 10.1016/j.bcp.2020.114279 33068552

[24] Menegon S Columbano A Giordano S. The Dual Roles of Nrf2 in Cancer. Trends Mol Med (2016) 22(7):578–93. 10.1016/j.molmed.2016.05.002 27263465

[25] Zhu B Tang L Chen S Yin C Peng S Li X. Targeting the Upstream Transcriptional Activator of PD-L1 as an Alternative Strategy in Melanoma Therapy. Oncogene (2018) 37(36):4941–54. 10.1038/s41388-018-0314-0 29786078

[26] Beury DW Carter KA Nelson C Sinha P Hanson E Nyandjo M. Myeloid-Derived Suppressor Cell Survival and Function Are Regulated by the Transcription Factor Nrf2. J Immunol (2016) 196(8):3470–8. 10.4049/jimmunol.1501785 PMC4821672 26936880

[27] Groth C Hu X Weber R Fleming V Altevogt P Utikal J. Immunosuppression Mediated by Myeloid-Derived Suppressor Cells (MDSCs) During Tumour Progression. Br J Cancer (2019) 120(1):16–25. 10.1038/s41416-018-0333-1 30413826 PMC6325125

[28] Song CH Kim N Nam RH Choi SI Kang C Jang JY. Nuclear Factor Erythroid 2-Related Factor 2 Knockout Suppresses the Development of Aggressive Colorectal Cancer Formation Induced by Azoxymethane/Dextran Sulfate Sodium-Treatment in Female Mice. J Cancer Prev (2021) 26(1):41–53. 10.15430/JCP.2021.26.1.41 33842405 PMC8020176

[29] Khor TO Huang MT Prawan A Liu Y Hao X Yu S. Increased Susceptibility of Nrf2 Knockout Mice to Colitis-Associated Colorectal Cancer. Cancer Prev Res (Phila) (2008) 1(3):187–91. 10.1158/1940-6207.Capr-08-0028 PMC3580177 19138955

[30] Li Y He M Zhou Y Yang C Wei S Bian X. The Prognostic and Clinicopathological Roles of PD-L1 Expression in Colorectal Cancer: A Systematic Review and Meta-Analysis. Front Pharmacol (2019) 10:139. 10.3389/fphar.2019.00139 30873025 PMC6403169

[31] Yang L Xue R Pan C. Prognostic and Clinicopathological Value of PD-L1 in Colorectal Cancer: A Systematic Review and Meta-Analysis. Onco Targets Ther (2019) 12:3671–82. 10.2147/ott.S190168 PMC6526188 31190869

[32] Chulkina M Beswick EJ Pinchuk IV. Role of PD-L1 in Gut Mucosa Tolerance and Chronic Inflammation. Int J Mol Sci (2020) 21(23):9165. 10.3390/ijms21239165 PMC7730745 33271941

[33] O'Malley G Treacy O Lynch K Naicker SD Leonard NA Lohan P. Stromal Cell PD-L1 Inhibits CD8+ T-Cell Antitumor Immune Responses and Promotes Colon Cancer. Cancer Immunol Res (2018) 6(11):1426–41. 10.1158/2326-6066.Cir-17-0443 30228206

[34] Rojo de la Vega M Chapman E Zhang DD. NRF2 and the Hallmarks of Cancer. Cancer Cell (2018) 34(1):21–43. 10.1016/j.ccell.2018.03.022 29731393 PMC6039250

[35] Neurath MF Becker C Barbulescu K. Role of NF-kappaB in Immune and Inflammatory Responses in the Gut. Gut (1998) 43(6):856–60. 10.1136/gut.43.6.856 PMC1727350 9824616

[36] Wang S Liu Z Wang L Zhang X. NF-kappaB Signaling Pathway, Inflammation and Colorectal Cancer. Cell Mol Immunol (2009) 6(5):327–34. 10.1038/cmi.2009.43 PMC4003215 19887045

[37] Botti G Fratangelo F Cerrone M Liguori G Cantile M Anniciello AM. COX-2 Expression Positively Correlates With PD-L1 Expression in Human Melanoma Cells. J Transl Med (2017) 15(1):46. 10.1186/s12967-017-1150-7 28231855 PMC5324267

[38] Markosyan N Chen EP Evans RA Ndong V Vonderheide RH Smyth EM. Mammary Carcinoma Cell Derived Cyclooxygenase 2 Suppresses Tumor Immune Surveillance by Enhancing Intratumoral Immune Checkpoint Activity. Breast Cancer Res (2013) 15(5):R75. 10.1186/bcr3469 24004819 PMC3979159

[39] Folkerd EJ Dowsett M. Influence of Sex Hormones on Cancer Progression. J Clin Oncol (2010) 28(26):4038–44. 10.1200/jco.2009.27.4290 20644089

[40] Lin H Wei S Hurt EM Green MD Zhao L Vatan L. Host Expression of PD-L1 Determines Efficacy of PD-L1 Pathway Blockade-Mediated Tumor Regression. J Clin Invest (2018) 128(2):805–15. 10.1172/jci96113 PMC5785251 29337305

[41] Tang H Liang Y Anders RA Taube JM Qiu X Mulgaonkar A. PD-L1 on Host Cells Is Essential for PD-L1 Blockade-Mediated Tumor Regression. J Clin Invest (2018) 128(2):580–8. 10.1172/jci96061 PMC5785245 29337303

