# Supplementary material for: The Enhanced Inhibitory Effect of Estrogen on PD-L1 Expression Following Nrf2 Deficiency in the AOM/DSS Model of Colitis-Associated Cancer
Source: Front Oncol. 2021 Jul 8;11:679324. doi: 10.3389/fonc.2021.679324 (PMC8297827; doi:10.3389/fonc.2021.679324)
Supplement: Supplementary file 1 [file DataSheet_1.docx]

Supplementary Material

The Enhanced Inhibitory Effect of Estrogen on PD-L1 Expression Following Nrf2 deficiency in the AOM/DSS Model of Colitis-Associated Cancer

**Changhee Kang, Chin-Hee Song, Nayoung Kim*, Ryoung Hee Nam, Soo In Choi, Jeong Eun Yu, Heewon Nho, Jin A Choi, Jin Won Kim, Hee Young Na, Ha-Na Lee and Young-Joon Surh**

*** Correspondence:** Nayoung Kim: nakim49@snu.ac.kr


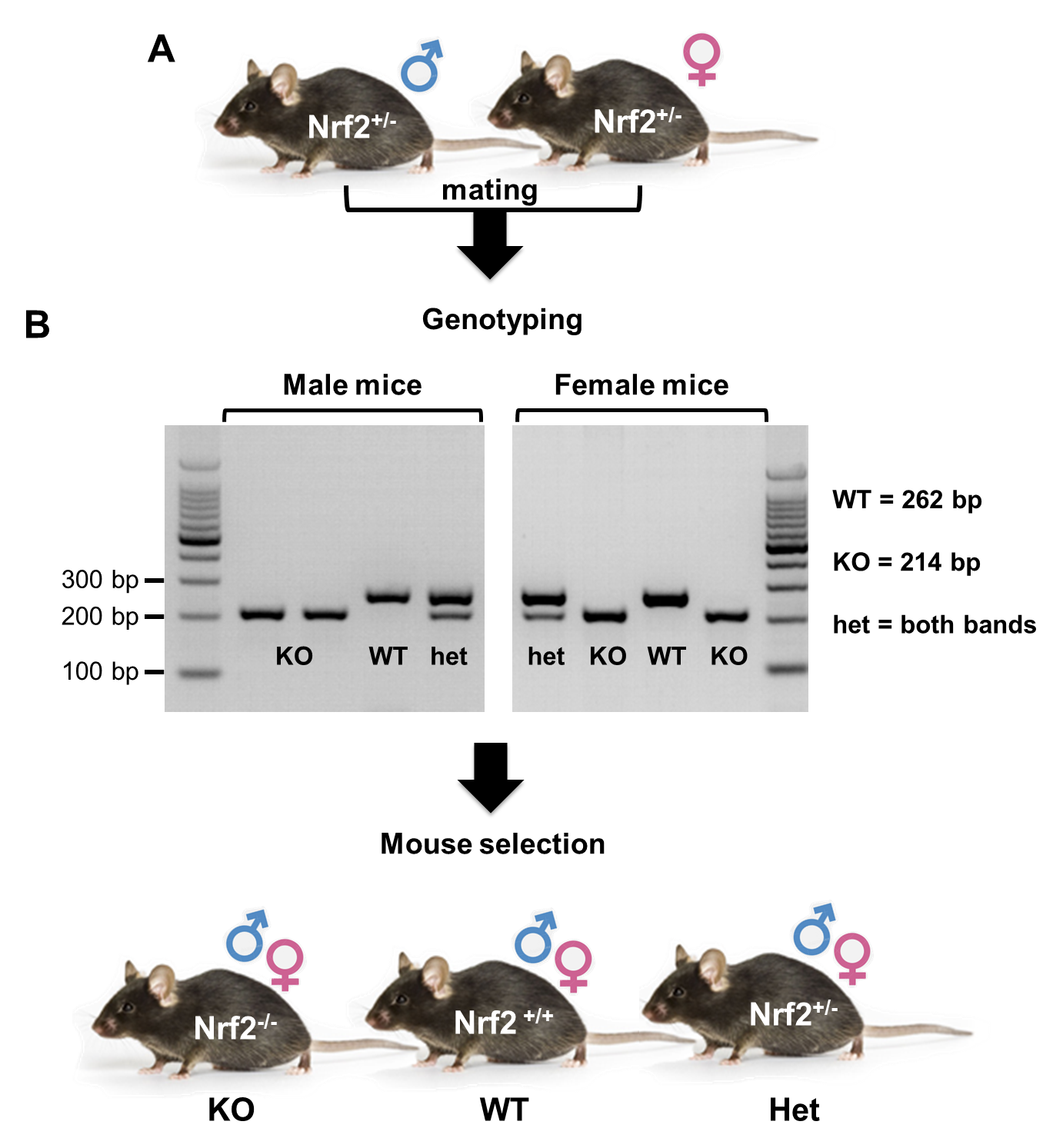


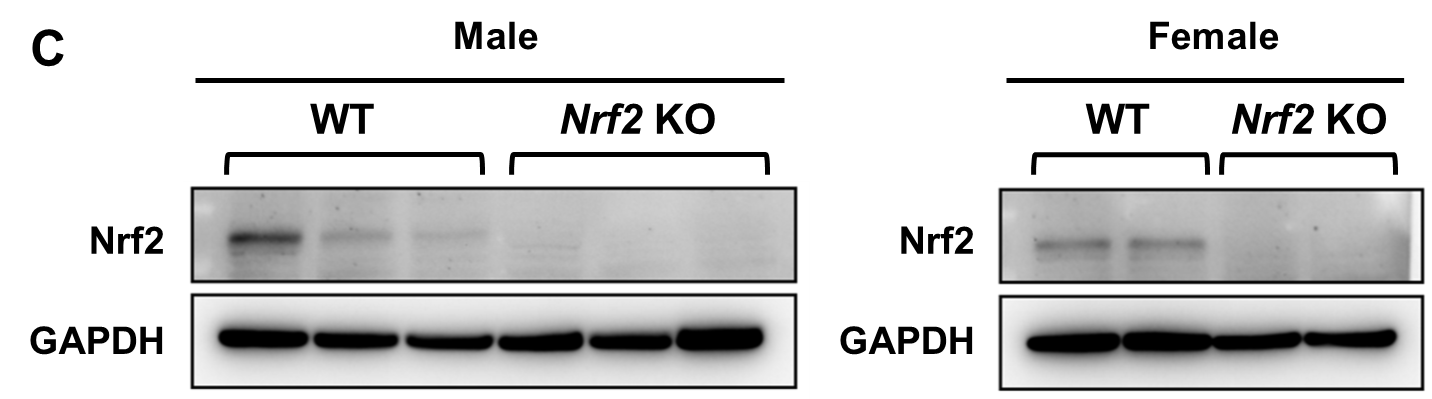


**Supplementary Figure S1.** Selection of WT and Nrf2 KO male and female mice using genotyping. (A) Heterogeneous Nrf2 KO (Nrf2^+/-^) male and female mice were mated. (B) Genotyping by PCR method. Representative agarose gel photos showing the PCR products of genomic DNA from tail of male and female mice. Nrf2 WT allele produces a 262-bp band, whereas the targeted allele produces a 214-bp band. Heterogeneous Nrf2 KO allele produces both, 214-bp and 262-bp bands. Selection of WT and homogeneous Nrf2 KO (Nrf2^-/-^) male and female mice (23, 29). (C) Validation of the Nrf2 protein expression in whole protein extracted from colon tissues of WT and Nrf2 KO male and female mice. ♂, male; ♀, female; +/+ and WT, wild-type; +/- and het, heterogeneous knockout; -/- and KO, homogeneous knockout

**Supplementary Reference**

23. Song CH, Kim N, Hee Nam R, In Choi S, Hee Son J, Eun Yu J, et al. 17β-Estradiol strongly inhibits azoxymethane/dextran sulfate sodium-induced colorectal cancer development in Nrf2 knockout male mice. Biochem Pharmacol (2020) 182:114279. doi: 10.1016/j.bcp.2020.114279

29. Song CH, Kim N, Nam RH, Choi SI, Kang C, Jang JY, et al. Nuclear factor erythroid 2-related factor 2 knockout suppresses the development of aggressive colorectal cancer formation induced by azoxymethane/dextran sulfate sodium-treatment in female mice. J Cancer Prev. 2021 Mar 30;26(1):41-53. doi: 10.15430/JCP.2021.26.1.41.
