# Supplementary material for: The Enhanced Inhibitory Effect of Estrogen on PD-L1 Expression Following Nrf2 Deficiency in the AOM/DSS Model of Colitis-Associated Cancer
Source: Front Oncol. 2021 Jul 8;11:679324. doi: 10.3389/fonc.2021.679324 (PMC8297827; doi:10.3389/fonc.2021.679324)
Supplement: Supplementary file 2 [file DataSheet_2.docx]

Supplementary Material

The Enhanced Inhibitory Effect of Estrogen on PD-L1 Expression Following Nrf2 deficiency in the AOM/DSS Model of Colitis-Associated Cancer

**Changhee Kang, Chin-Hee Song, Nayoung Kim*, Ryoung Hee Nam, Soo In Choi, Jeong Eun Yu, Heewon Nho, Jin A Choi, Jin Won Kim, Hee Young Na, Ha-Na Lee and Young-Joon Surh**

*** Correspondence:** Nayoung Kim: nakim49@snu.ac.kr


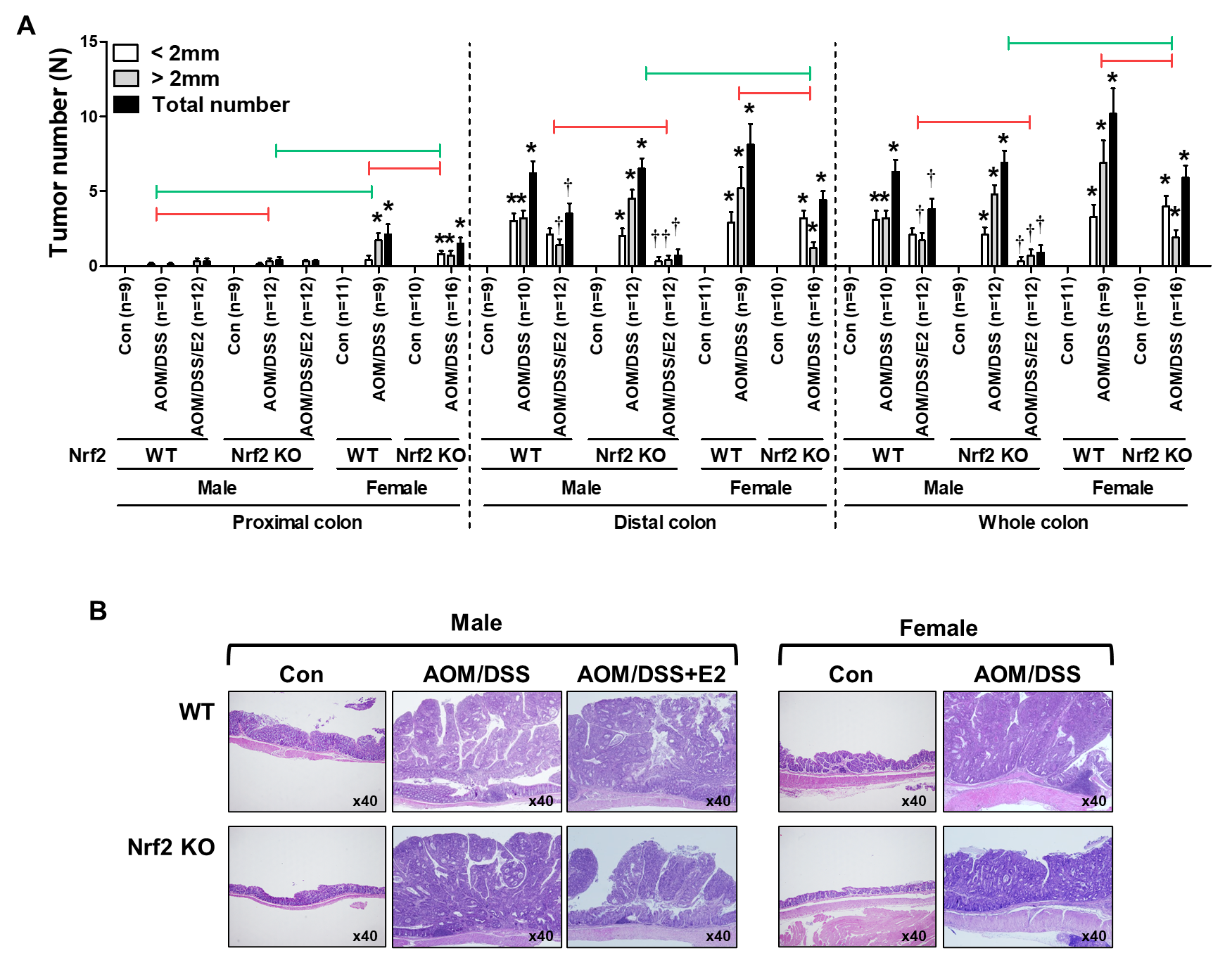


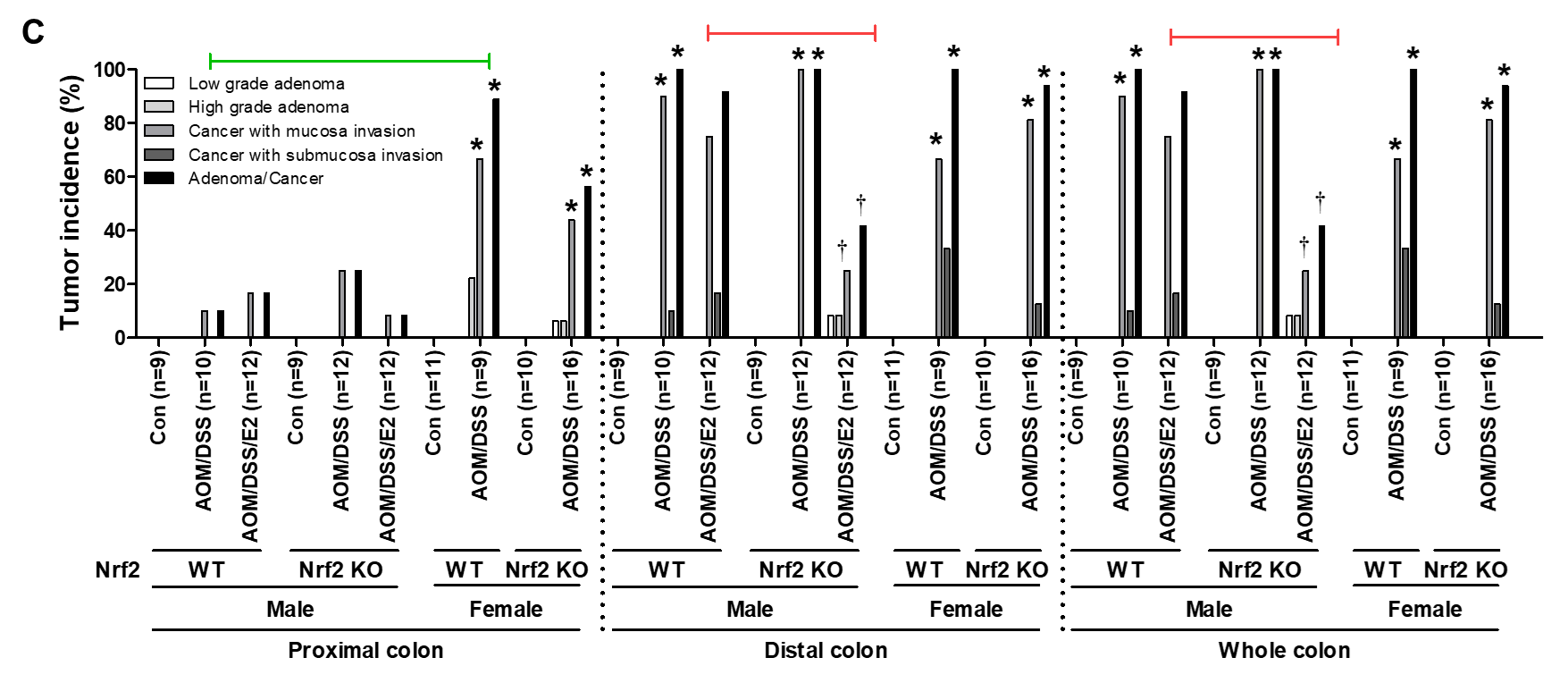


**Supplementary Figure S2.** Effect of E2 *via* Nrf2 on the colon tumor multiplicity at weeks 16. (A) Average tumor numbers and size distribution in the proximal, distal, and whole colon in each group sacrificed at week 16 following AOM injection. (B) Representative H&E staining images. Magnification, ×40. (C) Quantification of adenoma/adenocarcinoma incidence and invasion in each group by microscopic evaluation of the colonic tissues. Con, control male or mice; AOM/DSS, AOM and DSS-treated male or female mice; AOM/DSS+E2, AOM, DSS, and 17β-estradiol-treated male mice. *, *P* < 0.05 for Con. vs. AOM/DSS; †, *P* < 0.05 for AOM/DSS vs. AOM/DSS+E2; Green solid line, *P* < 0.05 for male vs. female; Red solid line, *P* < 0.05 for WT vs. Nrf2 KO. WT, wild-type; KO, knockout; AOM, azoxymethane; DSS, dextran sodium sulfate; E2, 17β-estradiol (23, 29).

**Supplementary Reference**

23. Song CH, Kim N, Hee Nam R, In Choi S, Hee Son J, Eun Yu J, et al. 17β-Estradiol strongly inhibits azoxymethane/dextran sulfate sodium-induced colorectal cancer development in Nrf2 knockout male mice. Biochem Pharmacol (2020) 182:114279. doi: 10.1016/j.bcp.2020.114279

29. Song CH, Kim N, Nam RH, Choi SI, Kang C, Jang JY, et al. Nuclear factor erythroid 2-related factor 2 knockout suppresses the development of aggressive colorectal cancer formation induced by azoxymethane/dextran sulfate sodium-treatment in female mice. J Cancer Prev. 2021 Mar 30;26(1):41-53. doi: 10.15430/JCP.2021.26.1.41.
